# Estimation of leisure time physical activity and sedentary behaviour among school adolescents in Nepal

**DOI:** 10.1186/1471-2458-14-637

**Published:** 2014-06-22

**Authors:** Susan Paudel, Narayan Subedi, Ramjee Bhandari, Ramesh Bastola, Rakshya Niroula, Amod Kumar Poudyal

**Affiliations:** 1 Maharajgunj Medical Campus, Institute of Medicine, Tribhuvan University, Kathmandu, Nepal; 2 Nepal Public Health Foundation, Kathmandu, Nepal; 3 Durham University, Durham, UK

**Keywords:** Cross-sectional study, Leisure time physical activity, Adolescents, Sedentary behavior, Nepal

## Abstract

**Background:**

Leisure-time physical activity is essential for healthy and physically active life; however, this domain of physical activity is less common in developing countries. Information on leisure time physical activity and sedentary behaviour among Nepalese population is not available. The study was carried out to assess leisure time physical activity and sedentary behaviour among high school adolescents and identify the associated factors in Nepal.

**Methods:**

A cross-sectional descriptive study was carried out in Banke district, Nepal in 2013 among higher secondary school students using self-administered questionnaire based on International Physical Activity Questionnaire. A sample of 405 students, 178 females and 227 males, of the age–group 15 to 20 years from seven schools were included in the study. Multivariate logistic regression analysis was carried out to identify factors associated with participation in leisure time physical activity and sedentary behaviour.

**Results:**

Engagement of female in leisure time physical activity was lower but mean time spent on sitting per day was higher. Students who walked to school and have playground/parks near home, younger females (OR = 3.09, 95% CI: 1.18-8.08), females living in nuclear families (OR: 2.16, 95% CI: 1.01-4.62) and males who cycled to school (OR: 8.09, 95% CI: 2.35-27.80) and have provision of extra-curricular activities (OR: 2.49, 95% CI: 1.04-5.97) were more likely to be engaged in leisure time physical activity. On the other hand, students who did not have playground in school and lived in rural areas were more likely to sit for more than 6 hours a day. Likewise, male students of private school (OR: 6.41, 95% CI: 2.89-14.21), who used vehicle to reach school (OR: 5.90, 95% CI: 1.26-27.75) and have no provision of extra-curricular activities (OR: 2.98, 95% CI: 1.09-8.07) had longer sitting time.

**Conclusion:**

Difference in leisure time physical activity and sedentary behaviour was found among male and female school adolescents. Interventions are needed not only to promote leisure time physical activity but also to reduce sedentary behaviour among this group.

## Background

Physical activity is an essential aspect of a healthy lifestyle [1,2] and leisure-time physical activity (LTPA) is one of the most important components of total physical activity [3], others being occupation, transportation and home-based activities [4]. Regular LTPA controls diabetes mellitus and obesity, reduces hypertension, cardio-vascular diseases, some cancers and provides other health benefits [5]. LTPA are more common in developed countries while occupational, household and transport domains are the major contributors to total physical activity in developing countries [4].

The transition from childhood to adolescence has been identified as period of marked decline in physical activity, particularly amongst girls [6,7], though this period is the most appropriate time for adoption of physical activity behaviors [8]. Studies from developing countries have reported gender difference in physical activity level and types of activities among adolescents. Girls appear to be less physically active than boys across all age groups and it associates with a number of individual, interpersonal and environmental covariates [9,10]. On the other hand, time spent in sedentary activities like watching television, sitting either at school or home, reading, playing or working on computers is important as time spent in these activities limit the time available for physical activities [11]. These days, many young people are reluctant to do physical activities and instead choose sedentary activities for their leisure time [12].

In Nepal, adolescents comprise about 27% of total population and the proportion is in increasing trend [13]. About 17% of the Nepalese population lives in urban areas [14]. Average family size of the country is 4.9 with an average of 4.3 in urban, 5.0 in rural areas and in particular 5.3 members in the terai region. At national level, 51% of the households are within the reach to nearest paved road and 80% households are within the reach to nearest vehicle passable dirt road within 30 minutes. Overall, 72% of currently school/college enrolled Nepalese students attend public schools and 44% attend such schools in urban areas [15]. Literacy rate among 15–19 age group is 94% which is slightly higher for males (96%) than females (91%). Among the same age group, the dropout rate for higher education is 12% among males and 14% among females [13].

Limited studies are available on physical activity and its correlates in Nepal [16]. In addition, information on LTPA and sedentary behaviour among school adolescents is almost non-existent. This study was carried out to assess prevalence of LTPA and sedentary behaviour and their associated factors among high school adolescents.

## Methods

### Study area

This study was conducted in Banke district, one of the 75 districts of Nepal. It is a terai district of the mid-western development region, which is around 500 km far west of Kathmandu, the capital city. The study was carried out in Nepalgunj municipality of the district. Though the study area has been purposively selected, it represents a typical urban terai.

The country is divided into three ecological regions which run parallel from east to west: Mountain in the north, Hill in the centre and Terai in the south. The Terai region consists of low-lying plain fertile land and agriculture is the main source of economy because of which this region is also called the ‘grainary’ of Nepal. In addition, Terai is the most productive region of the country as it consists of majority of the country’s industries. The region, though consists of only 23% of the total area of the country, shelters 50% of the Nepalese population [14].

In Nepal, municipalities are considered as urban area while VDC as rural area. The study district consists of 46 Village Development Committees (VDCs) and one municipality. About 15% of population of the district lives in urban area and average family size is 5.7. All VDCs and municipality in the district have access to motorable road [17].

### Selection of study participants

A cross sectional descriptive study was carried among 415 students of grade 11 and 12 from randomly selected seven higher secondary schools which included 3 public and 4 private schools. Public schools are run by the government and have cheaper fees while private schools are run by individuals or institutions mostly for profit and usually have higher fees. Altogether 10 out of 19 higher secondary schools in the municipality were approached for data collection but permission was obtained only from 7 schools. From each sampled school, one section of either grade 11 or 12 was randomly selected. All students of the selected sections were included in the study. Exclusion criteria were developed for students with physical disability related to hands or legs but no such students were found. Altogether, 415 students filled the questionnaire but 10 forms were discarded because of missing data and hence 405 was the final sample subjected for further analysis. Response rate was 98% in the study.

### LTPA, sedentary behaviour and its measurement

In the study, LTPA was assessed using International Physical Activity Questionnaire – Long form (IPAQ-LF). Though significant over-reporting existed, IPAQ - LF has been found to have acceptable validity to measure participant’s physical activity level [18-20] and test-retest reliability coefficients were also acceptable between 0 and 8 days [21]. IPAQ-LF has been found to be suitable for measuring physical activity in developing countries also [22].

In order to measure sedentary behaviour, students were asked total number of hours they spent on sitting per day. This included time spent on reading at school or home, travelling on vehicles, watching television, playing video games, working or playing on computer, etc. but time spent on each of these activities separately was not asked in the study.

Questionnaire, which included questions on LTPA, sitting time, socio-demographic and environmental characteristics, were filled by students in their respective classroom under the supervision of researcher or research assistants and their teachers in September 2013. Students were oriented for an hour on activities to be included in LTPA and types of moderate, vigorous and sedentary activities before filling the questionnaire. Pictures of different activities and sports that could be done during leisure were shown to make them understand and minimize recall bias which was likely as the students had to remember activities carried out during seven days preceding the study. These pictures were developed based on ‘physical activity show cards’of World health Organization.

Ethical approval was obtained from Institutional Review Board, Institute of Medicine and written informed consent was obtained from each student before filling the questionnaire. Confidentiality of information was assured and ensured throughout the research process.

### Statistical analysis

For analysis of LTPA, students were classified as LTPA or No LTPA based on their self-report. Students who did at least any form of leisure time physical activity for more than 10 minutes in any day of a week were categorized as “LTPA” and who did not do such activities were categorized as “No LTPA”. Activities which were carried for at least 10 minutes at a time were only included in the study. Student’s total energy expenditure from leisure time activities was computed by multiplying duration of activities per day by number of days per week and metabolic equivalent (MET) values. The MET values used were 3.3 METs for walking, 4 METs for moderate and 8 METs for vigorous activities [23].

Difference in socio-demographic and environmental characteristics between males and females, further categorized as LTPA or No LTPA, was tested using Pearson chi-square test and p-value less than 0.05 was taken as significant while difference in mean sitting time between these groups was compared using independent t-test. Logistic regression analysis was carried separately for males and females to identify variables associated with LTPA. Bivariate logistic regression analysis in which single factor was entered in the analysis model was used to obtain unadjusted odds ratio (OR) and 95% confidence interval (CI). All the independent variables were then entered at the same time in multivariate logistic regression analysis to adjust the effect of confounding. Adjusted OR was calculated to measure the net effect size of variables. Logistic regression analysis was also used with sitting time as the dependent variable to identify factors associated with sitting time of more than 6 hours. Statistical Package for Social Sciences (SPSS 17.0.2, release March, 2009) was used for data analysis.

## Results

Table 1 shows socio-demographic and environmental characteristics and sitting time among male and female students stratified by LTPA. Mean age of students was 17 ± 1.2 years. Among 405 respondents, 67% did at least some form of LTPA for more than 10 minutes at a time which was 80% and 50% among males and females respectively. Among 207 students who did at least some form of LTPA, average time spent was 49 minutes per day. It was higher among male students (55 minutes) compared to females (38 minutes). In contrast to this, mean time spent on sitting per day was comparably higher among females than males. Statistically significant difference was found between LTPA and No LTPA groups among both sexes in mode of transport to school and presence of playground or parks near home while significant difference was observed in economic status among females only.

**Table 1:** Socio-demographic, environmental characteristics and sitting time: differences by LTPA and gender

**Characteristics**	**Female (n = 178)**			**Male (n = 227)**		
	**No LTPA (n = 89)**	**LTPA (n = 89)**	**p**	**No LTPA (n = 46)**	**LTPA (n = 181)**	**p**
Age of student (in years)						
15-17	67 (47)	76 (53)	0.090	29 (18)	129 (82)	0.279
18-20	22 (63)	13 (37)		17 (25)	52 (75)	
Place of residence						
Rural	39 (53)	35 (47)	0.543	20 (22)	71 (78)	0.599
Urban	50 (48)	54 (52)		26 (19)	110 (81)	
Family type						
Nuclear	59 (47)	67 (53)	0.187	30 (20)	119 (80)	0.946
Joint/Extended	30 (58)	22 (42)		16 (21)	62 (80)	
Economic status						
Low	22 (73)	8 (27)	0.004	24 (23)	81 (77)	0.589
Medium	26 (38)	43 (63)		13 (20)	53 (80)	
High	41 (52)	38 (48)		9 (16)	47 (84)	
Type of school						
Public	31 (48)	34 (52)	0.640	21 (22)	75 (78)	0.605
Private	58 (51)	55 (49)		25 (19)	106 (81)	
Mode of transport to school						
Walking	35 (40)	53 (60)	0.006	25 (22)	90 (78)	0.003
Cycling	31 (53)	27 (47)		10 (12)	76 (88)	
Vehicles	23 (72)	9 (28)		11 (41)	15 (58)	
Playground in school						
No	46 (55)	37 (45)	0.176	26 (26)	76 (74)	0.077
Yes	43 (45)	52 (55)		20 (16)	105 (84)	
Extra-curricular activities at school						
No	70 (70)	65 (48)	0.381	34 (18)	151 (82)	0.138
Yes	19 (44)	24 (56)		12 (29)	30 (71)	
Neighborhood walkability						
No	4 (31)	9 (69)	0.150	5 (21)	19 (79)	0.942
Yes	85 (51)	80 (49)		41 (20)	162 (80)	
Playground/parks near home						
No	56 (59)	39 (41)	0.011	31 (25)	91 (75)	0.038
Yes	33 (40)	50 (60)		15 (14)	90 (86)	
Sitting time						
≤ 6 hours	15 (47)	17 (53)	0.696	13 (17)	66 (83)	0.297
> 6 hours	74 (51)	72 (49)		33 (22)	115 (78)	
Mean ± SD (hours/day)	8.3 ± 2.1	7.7 ± 1.8	0.082	7.5 ± 2.4	7.3 ± 2.4	0.734

Note: figures in parenthesis denote percentage.

LTPA scores and contribution of walking, moderate and vigorous activities among males and females is presented in Table 2. Median MET-minutes/week gained by the students from LTPA was 998 which was higher among males than females (1314 versus 678). Total LTPA score was contributed by walking (45%) followed by moderate (32%) and vigorous activities (23%). Engagement of males in vigorous activities was double than females (28% versus 14%) while engagement of females was higher in leisure time walking and moderate activities.

**Table 2:** LTPA scores by gender

**Characteristics**	**Total (n = 270)**			**Female (n = 89)**			**Male (n = 181)**		
	**Median MET-min/wk**	**Q**_**1**_ **~ Q**_**3**_	**% of total LTPA**	**Median MET-min/wk**	**Q**_**1**_ **~ Q**_**3**_	**% of total LTPA**	**Median MET-min/wk**	**Q**_**1**_ **~ Q**_**3**_	**% of total PA**
Total LTPA Score	998	480 ~ 2240		678	301 ~ 1608		1314	576 ~ 2589	
Activity specific PA scores									
Walking	297	99 ~ 594	45	198	0 ~ 544	49	396	115 ~ 693	43
Moderate	240	0 ~ 720	32	120	0 ~ 660	37	240	0 ~ 740	29
Vigorous	0	0 ~ 960	23	0	0	14	0	0 ~ 1360	28

Association between LTPA and socio-demographic, environmental and sitting time related variables is shown in Table 3. For both males and females, logistic regression analysis revealed that students who walked to school and students having parks or playgrounds near home were more likely to be engaged in LTPA. Younger (15–17 years) females were 3 times more likely to be engaged in LTPA than their older counterparts (OR = 3.09, 95% CI: 1.18-8.08). Similarly, females living in nuclear families were twice likely to be involved in LTPA than those living in joint or extended families (OR: 2.16, 95% CI: 1.01-4.62). On the other hand, female students from poor families were less likely to be engaged in such activities (OR: 0.34, 95% CI: 0.12-0.98). In case of male students, those having provision of extra-curricular activities at schools were nearly 2.5 times (OR: 2.49, 95% CI: 1.04-5.97) more likely to do LTPA than students having no provision of such activities whereas no association existed among females. Cycling to school was also associated among males only (OR: 8.09, 95% CI: 2.35-27.80). Place of residence, type of school, playground in school, neighborhood walkability and sitting time were not associated with LTPA among both sexes.

**Table 3:** Association between LTPA and socio-demographic, environmental characteristics and sitting time by gender

**Characteristics**	**Female**			**Male**		
	**Unadjusted**	**Adjusted**	**p**	**Unadjusted**	**Adjusted**	**p**
	**OR (95% CI)**	**OR (95% CI)**		**OR (95% CI)**	**OR (95% CI)**	
Age of student (in years)						
15-17	1.92 (0.89-4.10)	3.09 (1.18-8.08)	0.022	1.45 (0.74-2.87)	1.35 (0.62-2.95)	0.457
18-20	-	-		-	-	
Place of residence						
Rural	0.83 (0.46-1.51)	0.85 (0.36-2.04)	0.721	0.84 (0.44-1.61)	0.58 (0.24-1.36)	0.206
Urban	-	-		-	-	
Family type						
Nuclear	1.55 (0.81-2.97)	2.16 (1.01-4.62)	0.046	1.02 (0.52-2.02)	1.18 (0.55-2.54)	0.672
Joint/Extended	-	-		-	-	
Economic status			0.003			0.579
Low	0.39 (0.15-0.98)	0.34 (0.12-0.98)	0.046	0.65 (0.27-1.51)	0.64 (0.23-1.80)	0.402
Medium	1.78 (0.92-3.44)	2.14 (0.94-4.83)	0.069	0.78 (0.31-1.99)	0.99 (0.34-2.85)	0.982
High	-	-		-	-	
Type of school						
Public	1.16 (0.63-2.13)	1.75 (0.80-3.81)	0.161	0.84 (0.44-1.61)	0.69 (0.30-1.59)	0.382
Private	-	-		-	-	
Mode of transport to school						
Walking	3.87 (1.60-9.33)	3.82 (1.39-10.46)	0.009	2.64 (1.07-6.46)	3.74 (1.18-11.88)	0.025
Cycling	2.22 (0.88-5.62)	2.52 (0.85-7.42)	0.095	5.57 (2.01-15.45)	8.09 (2.35-27.80)	0.001
Vehicles	-	-		-	-	
Playground in school						
No	0.66 (0.36-1.20)	0.49 (0.23-1.06)	0.070	0.56 (0.29-1.07)	0.47 (0.20-1.11)	0.085
Yes	-	-		-	-	
Neighborhood walkability						
No	2.39 (0.71-8.07)	1.91 (0.47-7.84)	0.366	0.96 (0.34-2.73)	0.98 (0.31-3.13)	0.978
Yes	-	-		-	-	
Playground/parks near home						
No	0.46 (0.25-0.83)	0.48 (0.24-0.94)	0.034	0.49 (0.25-0.97)	0.42 (0.19-0.95)	0.038
Yes	-	-		-	-	
Extra-curricular activities						
Yes	0.73 (0.36-1.46)	0.82 (0.34-1.99)	0.662	1.77 (0.83-3.82)	2.49 (1.04-5.97)	0.041
No	-	-		-	-	
Sitting time						
≤ 6 hours	1.16 (0.54-2.50)	0.90 (0.35-2.31)	0.831	1.46 (0.71-2.96)	1.82 (0.70-4.70)	0.219
> 6 hours	-	-		-	-	

Note: Adjusted for age, place of residence, family type, economic status, type of school, mode of transport to school, playground and extra-curricular activities in school, neighborhood walkability, playground/parks near home and sitting time.

Logistic regression analysis between sitting time and independent variables presented in Table 4 showed that students living in rural areas and students who did not have playground in school were likely to sit for more than 6 hours a day. Male students of private school were six times more likely to sit longer hours than those studying in public school (OR: 6.41, 95% CI: 2.89-14.21). Similarly, male students who used vehicles to reach school (OR: 5.90, 95% CI: 1.26-27.75) were also likely to sit for more than 6 hours per day but no such association existed for female students. Females who cycled to school were less likely to sit for longer hours (OR: 0.14, 95% CI: 0.04-0.45). No provision of extracurricular activities increased the likelihood of sitting for longer hours among male students by nearly 3 times (OR: 2.98, 95% CI: 1.09-8.07) but had no effect on females. Similarly, no significant association was found with age, family type, economic status, neighborhood walkability and presence of parks or playground near home for both male and female students.

**Table 4:** Association between sedentary behaviour and socio-demographic, environmental characteristics and sitting time by gender

**Characteristics**	**Female**			**Male**		
	**Unadjusted**	**Adjusted**	**p**	**Unadjusted**	**Adjusted**	**p**
	**OR (95% CI)**	**OR (95% CI)**		**OR (95% CI)**	**OR (95% CI)**	
Age of student (in years)						
15-17	0.93 (0.35-2.47)	0.49 (0.13-1.79)	0.282	0.99 (0.55-1.81)	0.61(0.27-1.37)	0.232
18-20	-	-		-	-	
Place of residence						
Rural	3.03 (1.23-7.44)	4.82 (1.39-16.67)	0.013	5.66 (2.88-11.13)	5.55 (2.82-13.51)	<0.001
Urban	-	-		-	-	
Family type						
Nuclear	0.77 (0.32-1.85)	0.65 (0.23-1.83)	0.415	0.76 (0.42-1.36)	0.66 (0.31-1.41)	0.280
Joint/Extended	-	-		-	-	
Economic status						
Low	1.77 (0.47-6.72)	3.26 (0.68-15.56)	0.079	0.87 (0.44-1.72)	1.61 (0.62-4.19)	0.326
Medium	0.65(0.29-1.47)	0.84 (0.29-2.44)	0.138	1.10 (0.52-2.35)	1.42 (0.54-3.74)	0.478
High	-	-		-	-	
Type of school						
Private	1.69 (0.78-3.67)	2.37 (0.83-6.76)	0.105	4.55 (2.54-8.16)	6.41 (2.89-14.21)	<0.001
Public						
Mode of transport to school						
Vehicles	1.68 (0.44-6.31)	0.76 (0.16-3.53)	0.729	5.69 (1.62-20.04)	5.90 (1.26-27.75)	0.025
Cycling	0.46 (0.20-1.04)	0.14 (0.04-0.45)	0.001	1.62 (0.90-2.92)	1.10 (0.52-2.39)	0.791
Walking	-	-		-		
Playground in school						
No	4.42 (1.86-10.51)	4.53 (1.62-12.68)	0.004	5.93 (3.24-10.85)	3.66 (1.67-8.05)	0.001
Yes	-	-		-	-	
Extra-curricular activities						
No	0.78 (0.33-1.83)	1.55 (0.48-5.02)	0.468	1.64 (0.77-3.47)	2.98 (1.09-8.07)	0.032
Yes	-	-		-	-	
Neighborhood walkability						
No	0.36 (0.04-2.88)	0.22 (0.02-3.04)	0.259	2.45 (1.04-5.77)	1.89 (0.62-5.81)	0.263
Yes	-	-		-	-	
Playground/parks near home						
No	0.45 (0.21-1.01)	0.41 (0.15-1.06)	0.066	1.82 (1.04-3.18)	1.04 (0.46-2.32)	0.931
Yes	-	-		-	-	

Note: Adjusted for age, place of residence, family type, economic status, type of school, mode of transport to school, playground and extra-curricular activities in school, neighborhood walkability, playground/parks near home.

## Discussion

This cross-sectional study carried out to assess LTPA and sedentary behaviour among high school adolescents in Banke district of Nepal found that only two-third of the adolescents were involved in some form of LTPA. This could possibly be due to unavailability of parks and playgrounds for leisure time activities in Nepal [16] which may be further aggravated by unsafe roads [24] and less friendly urban environment. Studies indicate absence of aesthetic appeal and green spaces discourage active commuting and participation in leisure time physical activities [4]. In Nepal, children are expected to help their parents in income generating activities like agriculture or business and in addition, daughters are expected to take care of household chores which decrease their leisure time. Since more than half of the students were either from families whose main source of income was agriculture or business, engagement of students from such families in LTPA might have been lower. Adolescent and youth survey in Nepal has found that every 4 in 10 adolescents of the age group 15–19 are economically active [13]. On average, students spent 49 minutes per day on LTPA which is higher than found by another study in Nepal [25].

While considering sex of the student, engagement of females in LTPA was much lower as compared to males and female students spend less time on physical activities during leisure which is also supported by other studies [25]. Non-communicable disease risk factor survey conducted in 2007/8 and 2013 in Nepal have shown that females were less engaged in LTPA than their male counterparts [25,26]. Moreover, they were mostly engaged in leisure time walking and moderate activities which could possibly because in a country like Nepal, females tend to bear the burden of household works [16] and face greater restriction for outdoor movement while males mostly take responsibility of outdoor works which are supposed to be more vigorous. Daughters are expected to help their mothers with kitchen works while sons usually are allowed sufficient leisure time for playing with their friends [3,16]. A study in Nepal has found that mobility rate for male is three times higher than for females [13]. Other studies have also shown greater engagement of males in leisure time and vigorous activities [27-34].

In relation to sedentary behaviour, on average the students spent 7–8 hours per day on sitting while the NCD risk factor survey has shown average sitting time to be 2.5 hours among 15–29 age groups [25]. Students usually spent about 4–5 hours at school, mostly sitting, which might have contributed to longer sedentary time as found in this study. Furthermore, longer sitting time could also be attributed to increased use of modern technical gadgets among youths in urban areas of the country which are likely to replace physical activities during leisure [16].

Females in this study were not only just less engaged in LTPA, but also were likely to be more sedentary which indicated they spent their leisure time watching television, socializing or gossiping. Other studies have also shown females to be more sedentary [6,28,29,35,36] but Iranian females were less sedentary than their male counterparts [9]. Gender was not associated with sitting time in a study conducted among Polish students [37], these differences can be justified on grounds of social and cultural differences.

### Correlates of LTPA

Younger females were more likely to be engaged in LTPA as shown in the study while age was not a significant contributor among males. This could be because with the increase in age, female’s responsibility towards household chores as well as family restriction for spending longer hours with friends outside home increases and thus they have less time and options for LTPA. However, a study conducted in Taiwan has found age to be positively associated with participation in LTPA [38]. Less domestic work burden and less restriction for outdoor activities for females in nuclear families might have contributed to greater engagement of female students living in nuclear families on LTPA as found in the study.

In contrast to the study among Spanish adolescents, which found that male students studying in public schools were less engaged in LTPA than those studying in private schools [31], this study found no association between LTPA and type of school for both male and female students. This might be due to similar availability of facilities for physical activity during breaks in public and private schools. Students having playground and provision of extra-curricular activities at school and those who had parks or playground near their home were more engaged in LTPA. Other studies also showed that access to physical activity spaces increases likelihood of being active, especially among girls [6]. This highlights possible intervention areas to promote LTPA among students.

Individuals living in town and those with low income were found to be less engaged in LTPA in Taiwan [39], however, no association existed in other similar studies [34,38]. Females from poor families were found to be less engaged in LTPA in this study which might be due to greater involvement of those females in household works as their parents might be involved in income generating activities outside home. As a result, they might have less time for recreational activities. Likewise, the study found no association between sitting time or sedentary behaviour in general, with participation in LTPA for both boys and girls, which is consistent to the findings of a longitudinal study among US adolescents [40]. This indicates participation in sedentary activities and LTPA are “separate constructs, not functional opposites” [34,40,41] while another study has revealed sedentary behaviours displace physical activities among girls but not among boys [6].

### Correlates of sitting time

Studies have shown higher sedentary behaviour, particularly screen time, among rural adolescents [7], which is consistent to this study. Students residing in urban areas were likely to sit fewer hours per day than those residing in rural areas. This could be explained because the students from rural areas mostly use vehicles or cycles to reach school. Sitting time as studied also includes the time spent on vehicles. For those students using cycles, rest time at home could also be longer due to tiredness. In contrast to this, higher sedentary behaviour among urban residents has also been reported [42,43]. Furthermore, this study has also found higher sitting time among male students using vehicles to reach to school.

Female students who cycled to school spent less time on sedentary activities than those who used vehicles. This finding is congruent with the study conducted in North East England [36]. Furthermore, male students at private school had higher sitting time than those at public schools. A possible explanation could be longer study hours, regular class and sometimes extra classes at private school which increase overall time spent on sitting. On the other hand, public schools in Nepal have comparatively shorter school hours, longer breaks and irregular classes.

Results showed no association between age of student and sitting time. However, a study conducted among German adults has shown a reduction in sitting time with increasing age for both genders [44] while other studies have shown an increase in sedentary time with age [35,45,46]. Studies have also reported that children and adolescents of low socio-economic status have greater engagement in sedentary behaviours [47] and socio-economic status influences type of sedentary activities but may not have any impact on overall sedentary behaviour [45]. Students of lower socio-economic class spend more time on screen-based sedentary activities [35,48] while those of higher socio-economic class spend more time on academic sedentary activities [35]. Economic status was not associated with sitting time in this study and other studies [44,49].

Based on researcher’s observation, in Nepalese context, physical activity, especially LTPA is not a priority issue at community as well as at schools and most people are unaware about the risks of physical inactivity.

### Strengths and limitations of the study

Schools and participants of the study have been selected randomly which increases the strength of the study. It uses a reasonably large sample (n = 405) and the study has examined socio-demographic and environmental determinants of LTPA and sedentary behaviour which have been further segregated according to gender. This provides a better picture of gender difference in LTPA and sedentary behaviour and their determinants in Nepal.

Overall time spent on sitting is taken as a proxy indicator of sedentary behavior in the study. It is further not differentiated for weekdays and weekends. The study gives the cumulative time spent on sitting either at school or home but does not give information on time spent on different screen-based or academic sedentary activities. The study findings are based on self report of the students because of which findings are likely to suffer from over-reporting and recall bias which is another possible limitation; though measures were taken to address it. Although IPAQ has been used to measure physical activity in developed and developing countries including Nepal, its validity and reliability has not been examined in Nepal which can be a possible limitation of the study. Furthermore, cross-sectional nature of the study limits drawing inferences about causation.

## Conclusion

The study highlights gender difference in LTPA and sitting time. Females were found to spend less time in LTPA but more time on sitting. LTPA and sedentary behaviour were found to be two separate behaviours as no association existed between sitting time and LTPA in the study. Hence, specific interventions are needed not only to promote LTPA but also to reduce sedentary behaviour among school adolescents. Further studies using both subjective as well as objective measures for assessing LTPA and sedentary behaviour considering time spent on different domains and activities are recommended at school setting.

## Abbreviations

IPAQ-LF: International Physical Activity Questionnaire-Long form; LTPA: Leisure-time physical activity; MET: Metabolic Equivalent; VDC: Village Development Committee.

## Competing interests

The authors declare that they have no competing interests.

## Authors’ contributions

SP has a critical role in conceptualizing the study, designing the research protocol, developing questionnaire and in carrying out all aspects of this research including data collection, analysis and preparing manuscript. NS guided in conceptualizing the study, directed and supported to carry out the study and contributed in writing the manuscript especially the methodology. RN and RBs reviewed the manuscript and provided their comments before finalization. AKP supported in statistical analyses, interpretation of data and critically reviewed the manuscript. All authors read and approved the final manuscript.

## Authors’ information

SP, RN, RB: MPH graduate, Maharajgunj Medical Campus, Institute of Medicine, Tribhuvan University, Nepal

NS: Program Manager, Nepal Public Health Foundation

RB: MA candidate, MA- Risk, Health and Public Policy, Department of Geography, Durham University, Durham, UK

AKP: Associate Professor, Department of Community Medicine and Public Health, Maharajgunj Medical Campus, Institute of Medicine, Tribhuvan University, Nepal

## Pre-publication history

The pre-publication history for this paper can be accessed here:

http://www.biomedcentral.com/1471-2458/14/637/prepub
